# Mitogen-Activated Protein Kinase and Substrate Identification in Plant Growth and Development

**DOI:** 10.3390/ijms23052744

**Published:** 2022-03-02

**Authors:** Min Jiang, Youze Zhang, Peng Li, Jinjing Jian, Changling Zhao, Guosong Wen

**Affiliations:** 1 Ministry of Education Key Laboratory for Biodiversity Science and Ecological Engineering, Institute of Biodiversity Science, School of Life Sciences, Fudan University, Shanghai 200438, China; 19110700002@fudan.edu.cn; 2 State Key Laboratory of Tea Plant Biology and Utilization, Anhui Agricultural University, Hefei 230036, China; zhangyouze209@stu.ahau.edu.cn; 3 Shanghai Key Laboratory of Plant Functional Genomics and Resources, Shanghai Chenshan Botanical Garden, Shanghai 201602, China; lipeng@csnbgsh.cn; 4 Research & Development Center for Heath Product, College of Agronomy and Biotechnology, Yunnan Agricultural University, Kunming 650201, China; zhaoplumblossom7@ynau.edu.cn (C.Z.); wengs@ynau.edu.cn (G.W.)

**Keywords:** MAPK cascades, signal transduction, substrates, abiotic stress, biotic stress, docking site

## Abstract

Mitogen-activated protein kinases (MAPKs) form tightly controlled signaling cascades that play essential roles in plant growth, development, and defense response. However, the molecular mechanisms underlying MAPK cascades are still very elusive, largely because of our poor understanding of how they relay the signals. The MAPK cascade is composed of MAPK, MAPKK, and MAPKKK. They transfer signals through the phosphorylation of MAPKKK, MAPKK, and MAPK in turn. MAPKs are organized into a complex network for efficient transmission of specific stimuli. This review summarizes the research progress in recent years on the classification and functions of MAPK cascades under various conditions in plants, especially the research status and general methods available for identifying MAPK substrates, and provides suggestions for future research directions.

## 1. Introduction

The mitogen-activated protein kinase (MAPK and MPK) family is the largest group of transferases in eukaryotes, phosphorylating corresponding serine/threonine (Ser/Thr) residues, which are activated by both environmental and developmental signals [1,2]. The MAPK cascade pathway involves three kinases: mitogen-activated protein kinase kinase kinases (MAPKKKs, MKKKs, MAP3Ks, and MEKKs), mitogen-activated protein kinase kinases (MAPKKs, MKKs, and MAP2Ks), and MAPKs. In general, extracellular signals activate MAP3Ks, which, in turn, phosphorylate and activate the S/T-X_3-5_-S/T motif of downstream MAPKKs, and then, the activated MAPKKs also phosphorylate and activate MAPKs at their TXY activation loop and transfer the signal into the nucleus [3,4]. Therefore, highly ordered protein–protein interactions (PPIs) are the basis of MAPK-mediated signal transduction.

Since the first *MAPK* gene was identified in the model plant *Arabidopsis thaliana* in 1993, many MAPK cascades have been identified in *A. thaliana*, rice, *Brachypodium distachyon*, tobacco, and other plants [5,6,7]. In theory, these kinases can form different combinations involved in multiple biotic and abiotic stresses as well as cell division and development. However, the genetic evidence for random MAPK cascade combinations remains elusive [1,8]. In addition, a comparative study of the evolutionary history of the MAPK gene found that the specific cascade is a limited combination retained in the evolutionary process [9]. 

As is well-known, the research and understanding of MAPK cascade substrates in plants are limited. Genetic interaction methods are generally used to identify MAPK cascades [10], and large-scale screening methods have also been established [11,12]. Moreover, the interaction between MAPK and its substrate was verified by various kinase experiments, and the properties of MAPK as a proline-oriented Ser/Thr kinase were used to predict the substrate [13]. Recently, mass spectrometry-based phosphoproteomics has been used for the identification of plant kinase substrates [14,15,16], and great progress has been made in the MAPK cascade regulation of plant development and adaptation to environmental changes. In addition, computational methods based on evolutionary information, protein structure, physical and chemical characteristics, and 3D structural energy values have also been applied to the identification of plant kinase substrates [17]. The role in plant signal transduction has attracted more and more attention with the further study of the MAPK pathway.

## 2. Composition and Classification of MAPK Cascades in Plants

**Figure 1:**
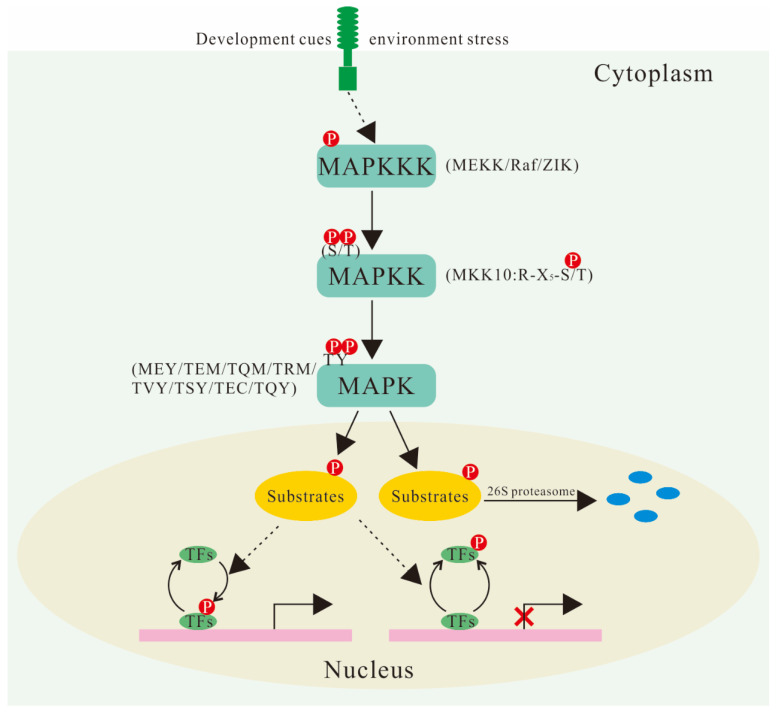
Schematic representation of MAPK cascade. A signal transduction cascade navigates the signal from MAPKKK to MAPK by triggering a series of Thr/Tyrosine (Tyr) and Ser/Thr phosphorylation events. Eventually, the activated MAPK is transported to the nucleus, where it is involved in the phosphorylation of transcription factors (TFs) and reconfigures a specific response related to transcriptional reprogramming. Phosphorylated substrates can be degraded by 26S proteasome or activated, and they can thus change the binding affinity with the promoter of the target gene to suppress or promote its expression. MAPKKK can fall into three subfamilies: MEKK, Raf (Rapidly Accelerated Fibrosarcoma), and ZIK (ZR1-interacting kinase). Compared with typical MKKs, the active loop of MKK10 displayed only a partially conserved Ser/Thr site (R-X_5_-S/T). The phosphorylation sites of MAPKs at their TXY activation loop include various forms, such as TEY (Thr-Glu-Tyr), TDY (Thr-Asp-Tyr), MEY (Met-Glu-Tyr), TEM (Thr-Glu-Met), TQM (Thr-Gln-Met), TRM (Thr-Arg-Met), TVY(Thr-Val-Tyr), TSY (Thr-Ser-Tyr), TEC (Thr-Glu-Cys), and TQY (Thr-Gln-Tyr). The circled P indicates phosphorylation; the blue circle indicates degraded protein.

In the *A. thaliana* genome, 20 coding *MAPK* genes have been identified and can be divided into four subfamilies (A–D) according to their characteristics and phylogenetic relationships (Table 1). In addition to TEY (Thr-Glu-Tyr) and TDY (Thr-Asp-Tyr), the conserved phosphorylation sites also include MEY (Met-Glu-Tyr), TEM (Thr-Glu-Met), TQM (Thr-Gln-Met), TRM (Thr-Arg-Met), TVY(Thr-Val-Tyr), TSY (Thr-Ser-Tyr), TEC (Thr-Glu-Cys), and TQY (Thr-Gln-Tyr), according to the comparison of MAPK sequences in different plants (Figure 1) [6]. Many *MAPK* genes have been identified from higher plants, most of which have high homology with the mammalian MAP kinase ERK family. The interaction between protein kinases is usually achieved by docking the interacting sites or scaffold proteins [18]. The C-terminal of MAPK proteins contains an evolutionally conserved common docking (CD) site, which acts as a docking site (D-site) for MKK, phosphatase, and substrate proteins. Ichimura et al. integrated identified MAPKs (Arabidopsis, alfalfa, tobacco, rice and barley, etc.) and developed a taxonomic nomenclature [19]. However, Mohanta et al. proposed a more complete taxonomic nomenclature, which provided a reference for the classification and nomenclature of newly identified MAPKs in other species on the basis of the diversity of 589 conserved MAPK sequences and variable differences of active loops in 40 plant species [6].

There are ten genes encoding MKKs in the Arabidopsis genome divided into four groups (A–D), only half as many as the number of MAPKs, suggesting that one MKK can activate different MAPKs. Hence, the cross-network of various signal transduction pathways is mainly concentrated at this level in plant MAPK cascades. However, phylogenetic analysis of MKKs in 51 plants suggested that the previous group D (MKK7/8/9/10) should be subdivided into group D (MKK7/8/9) and Group E (MKK10) (Table 1) [20]. The active loop of MKK10 showed only partially conserved motifs (R-X_5_-S/T) (Figure 1). Although the active loop of Group E MKK10 is incomplete, it still has a biological function. For example, both *mkk10/mpk3* and *mkk10/mpk6* double mutants had higher root growth rates than either single mutant [23]. MKK10 is involved in the rice defense response and maize (*Zea mays*) ethylene (ET)-mediated cell death and can phosphorylate MPK3 and MPK6 in vivo [24,25]. In addition, MKK10 paralogs from ancient tandem duplicates may be responsible for functional divergence in monocots, such as *B. distachyon* [26]. Moreover, the C-terminal region of MKK3 contains a specific nuclear transport signal peptide 2 (NTF2), which indicates that there may be a transport process from the cytoplasm to the nucleus during the response of the MKK family to stress. Further analysis showed that the gradual loss of MKK3’s cytoplasmic nucleus transport function may be related to more and more refined functions of plants during the evolution process [20,27].

It is predicted that there are 80 *MAPKKK* gene members in the Arabidopsis genome, which can be divided into three groups: MEKK (21 members), Raf (Rapidly Accelerated Fibrosarcoma; 48 members), and ZIK (ZR1-interacting kinase; 11 members), according to the analysis of amino acid correlation in the catalytic region of the protein kinase (Table 1 and Figure 1). Most *MEKK* gene members contain a typical conserved catalytic domain that activates downstream MKKs in response to stress. Raf consists of more than half of MAPKKKs and contains a specific polypeptide signal, GTxx(W/Y)MAPE [21]. Arabidopsis Constitutive Triple Response 1 (CTR1) and Enhanced Disease Resistance 1 (EDR1) are homologous to mammalian Raf MAPKKKs and are involved in ET and pathogen resistance-mediated signal transduction and the defense response, respectively. The plant ZIK subfamily, originally named ZIK1, is a predicted MAPKKK associated with the MPK regulatory protein ZR1. Although Raf and ZIK are typical MAPKKK family genes, their phosphorylation sites have not been confirmed in plants [28]. However, they have shown an ability to interact with MKKs in *Brassica napus* [29]. 

## 3. MAPK Cascades Are Involved in a Variety of Signal Transduction Pathways in Plants

### 3.1. MAPK Cascades in ROS Signaling

**Figure 2:**
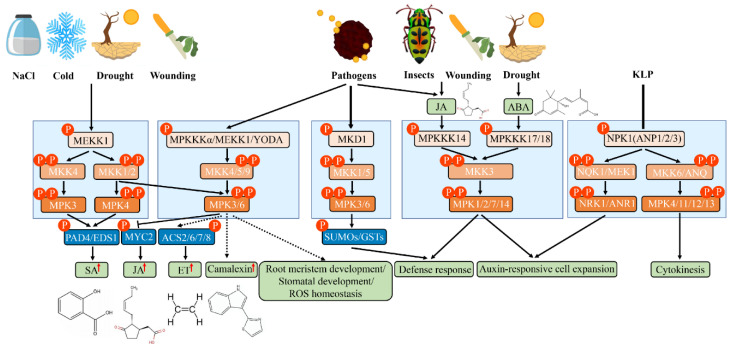
Overview of the functions of different MAPK cascades in stress responses as well as their regulation and possible downstream consequences. Upon sensing the tissue/cell-specific input signaling molecules, the MAPK cascade is activated in a spatiotemporal-specific manner. Activated MAPKs phosphorylate their substrate(s), ultimately leading to tissue-specific cell proliferation or differentiation at specific developmental stages or in response to specific external stimuli, thereby regulating plant growth, development, and stress responses. Arrows with uninterrupted lines indicate interactions supported by genetic and/or biochemical evidence, while arrows with dashed lines represent putative signaling pathways. One arrow may represent multiple steps because of unknown components in the signaling pathways. PAD4, Phytoalexin Deficient 4; EDS1, Enhanced Disease Susceptibility 1; ACS, 1-aminocyclopropane-1-carboxylic acid synthase; MKD1, MAPKKK δ-1; SUMO, small ubiquitin-like modifier; GST, glutathione S-transferase; ROS, reactive oxygen species; KLP, kinesin-like protein; NPK1, nucleus- and phragmoplast-localized protein kinase 1; ANP, Arabidopsis NPK1-related protein kinases; SA, salicylic acid; JA, jasmonic acid; ET, ethylene; ABA, abscisic acid. See text for details.

One of the ways that plants respond to environmental stress is to activate the MAPK pathway by producing reactive oxygen species (ROS) [30], and the identification of the initial site of ROS generation is the premise of studying this signal transduction event. When plants respond to stresses, such as pathogen or mechanical damage, ROS are first produced in the extracellular body [31], but the production sites in the chloroplasts, peroxisomes, or mitochondria have not been identified [32]. There are data that suggest that different ROS-dependent MPK signaling pathways are activated in plants in response to environmental stress. The OXI1 (oxidative stress inducible 1)-MPK3/6 signaling pathway is activated by ROS treatment in *A. thaliana* [33]. After H_2_O_2_ treatment, the MPK4 activity of Arabidopsis *mekk1* mutants decreased, while the MPK3, MPK4, and MPK6 activity increased. In addition, Medicago OMTK1 (oxidative stress-activated MAP triple-kinase 1), highly homologous to Arabidopsis MEKK1, is also activated by ROS induction [34]. It was reported that ROS accumulation induced by abscisic acid (ABA) activation can induce stomatal closure through calcium channels in the plasma membrane of guard cells in *A. thaliana* [35]. AIK1 (ABA-INSENSITIVE PROTEIN KINASE 1), induced by ABA, regulates the differentiation and elongation of Arabidopsis root cells and the stomatal response through the MKK5–MPK6 pathway [36]. NtMPK4-silenced tobacco (*Nicotiana tabacum*) is hypersensitive to ROS, suggesting that NtMPK4 may function in the early stages of ROS signaling by controlling ROS absorption. Arabidopsis YODA (YDA) can form a signaling pathway with its downstream kinases, MKK4/5 and MAPK3/6, to regulate stomatal phenotypes in epidermal cells (Figure 2) [37]. The NtMEK2-SIPK (salicylic acid (SA)-induced protein kinase) and NTMEK1-NTF6 (*Nicotiana* Fus-3-like kinase 6) signaling pathways can regulate the expression of the *NbRBOHB* (*Nicotiana benthamiana* Respiratory Burst Oxidase Homolog B) gene and the production of RBOH-dependent ROS. The potato (*Solanum tuberosum*) StMEK2 gene can also regulate cell death and ROS production caused by the hypersensitivity response (HR) by inducing *NbRBOHB* gene expression [38]. In addition, MPK8 can regulate the Ca^2+^ and ROS produced in response to early damage, avoid excessive ROS accumulation in cells, and maintain the balance of intracellular ROS [39]. Salt intolerance gene 1 (SIT1) can also induce ROS accumulation and activate MPK3 and MPK6 in rice, which are mainly expressed in root epidermal cells and induced by NaCl [40]. In other plant species, maize ZmMPK5 is also known to suppress ROS synthesis and ROS-induced damage, improving plant tolerance to cold stress [41], and wheat (*Triticum aestivum*) *TaMPK14* can modulate ROS homeostasis under N-starvation stress [42].

### 3.2. MAPK Cascade in Hormone Signaling

1-Aminocyclopropane-1-carboxylic acid synthase (ACS) is a rate-limiting enzyme for ET synthesis. AtMPK6 can inhibit the degradation of ACS2/6 by 26S proteasome and then promote ET synthesis (Figure 2) [43]. After DEX treatment, the *MKK4^DD^* and *MKK5^DD^* plants showed significant increases in MAPK6 and ACS activity [44], suggesting that the MKK4/5-MPK3/6-ACS2/6 pathway is involved in ET synthesis. In addition, the dephosphorylation of ABI1 (ABA insensitive 1; a protein phosphatase 2C (PP2C)) is involved in regulating the degradation of the ACS6 proteasome [45]. The *mkk9* mutant has a similar phenotype to the double mutant *ein3eil1* in the ET signal transduction pathway and is not sensitive to ET [46]. ET can activate the MKK9–MAPK3/MAPK6 pathway, which in turn activates the 174th amino acid of EIN (ETHYLENE INSENSITIVE 2) protein and keeps EIN3 stable. CTR1 can activate the 569th amino acid on the EIN protein through an unknown pathway, thereby degrading EIN3. Mechanical damage-induced ET synthesis is associated with MKK6–MPK3/6. Among them, ACS6 and ACS7 were rapidly induced 30 min after damage, while ACS2 and ACS8 reached their maximum value at 2–6 h later [47]. In addition, activated rose (Rosa hybrida) RhMPK6 phosphorylates and stabilizes RhACS1 and stimulates ET production in the pistils of roses. Further analysis found that RhMKK9 could regulate the expression and activity of RhMPK6 and RhACS1, suggesting that RhMKK9 is the activator of RhMPK6–RhACS1 [48].

Recent studies have found that MKK3 and MPK6 act as negative regulators of the jasmonic acid (JA) signal [49]; MPK1 and MPK2 can be activated by methylated JA (Me-JA) [50]. MPK7 and MPK14, belonging to subgroup C MAPKs, can interact physiologically with MKK3. In comparison with MPK6, the AtMKKK14–MKK3–MPK1/2/7/14 signaling pathway may play a major role in JA-dependent signaling [51], while AtMPK9 and AtMPK12 jointly participate in JA-induced stomatal closure (Figure 2) [52]. The induction of the transcriptional level of OsMPK7, OsMPK20-5, and OsMPK16 by JA treatment has been confirmed in rice [53]. Similarly, NtMPK1, NtMPK5, NtMPK8, NtMPK10, and NtMPK16 of tobacco and BdMPK7-1 and BdMPK20-5 of Brachypodium were induced after JA treatment [22,54]. 

MPK4 is involved in the regulation of the balance between the SA- and JA/ET-related defense response [55]. Enhanced disease susceptibility 1 (EDS1) and phytoalexin deficient 4 (PAD4) are the core antagonists between the SA and ET/JA defense signaling pathways to a certain extent, serving as positive regulators of SA accumulation and negative regulators of ET/JA signaling pathways. Specifically, *mpk4* mutants showed SA accumulation and sustained expression of the SA-related gene *PR* (pathogenesis-related), which regulated the expression of endoplasmic reticulum (ER)- and Me-JA-related genes. Interestingly, *eds1* and *pad4* mutants and MPK4-overexpressing transgenic plants can reduce SA content and partially recover the phenotype of *mpk4*. The double mutant also reduced the plant *dwarf* phenotype, reduced the SA content, and partially recovered the expression of the Me-JA-dependent *PLANT DEFENSIN1.2* (*PDF1.2*) gene. These results suggest that MPK4 regulates hormone balance in plants through EDS1/PAD4 (Figure 2) [55]. In the yeast two-hybrid (Y2H) system, MEKK1 interacts with MAPK4, and MEKK1 regulates defense-related hormone balance by regulating MAPK4. In addition, continuously activated MKK7 can cause SA accumulation, *PR1* gene continuous expression, and an increase in pathogen resistance [56], suggesting that there may be another MAPK cascade pathway antagonizing the effects of MPK4 in plants. The activity of AtMPK3 and AtEDR1 is regulated by SA, in which AtMPK3 functions as a negative regulator of SA accumulation induced by Flag22 [57]. Heterologous expression of inactive maize ZmMKK6 induces SA accumulation and SA-dependent leaf senescence in *A. thaliana* [58], and ZmMKK6 also activates ZmMPK4-1 and AtMPK4 in vitro. 

MKK7 positively regulates polar auxin transport (PAT), while the overexpression of MKK7 causes a lack of PAT, leading to malformation changes in *A. thaliana* [59]. Interestingly, PAT is regulated by PIN-FORMED proteins (PINs) and reversible protein phosphorylation, including protein kinases and protein phosphatases that mediate the activity of auxin transporters, such as MPK4 and MKK7/MPK6 modules [60]. AtMKK3–MPK1–RBK1 (ROP binding protein kinase 1) regulates auxin-dependent cell expansion [61]. MKKK17/18, a known upstream kinase of MKK3, is associated with ABA signaling [62], suggesting that the ABA-activated MKKK17/18–MKK3–MPK1/2/7/14 cascade is cross-talk between ABA and auxin signaling (Figure 2). Raf10 phosphorylates and enhances sucrose non-fermenting 1 (SNF1)-related protein kinase (SnRK2.3) activity and may facilitate its release from negative regulators in response to ABA signaling [63]. ZmMPK6 plays a role in ABA-induced antioxidant defense by phosphorylating ZmWRKY104 in maize [64]. 

### 3.3. MAPK Cascades in Biotic Stress

Higher plants have developed mechanisms to detect and rapidly respond to pathogen invasion using their own immune systems in the evolutionary process. There are two MAPK cascade pathways, MEKK1–MKK4/5–MAPK3/6–WRKY22/29 [65] and MEKK1–MKK1/2–MPK4 [66], activated by bacterial flagellin Flg22 in *A. thaliana*. Both *mpk4* and *mekk1* mutants had short stature, spontaneous cell death in leaves, and sustained expression of pathogen-related genes, such as *PR1* and *PDF1.2*, and showed resistance to pathogen infection [67]. Meanwhile, it was found that MPK4 could bind and phosphorylate its direct substrate MKS1 (MAP kinase 4 substrate 1), which could bind WRKY33 [11]. In conclusion, MEKK1 and MPK4 play an important role in perceiving and responding to Flag22 infection and negatively regulate the programmed cell death (PCD) process induced by pathogens or pathogen-associated molecular pattern (PAMP). Additionally, the acetylation of MKK7 also contributes to plant immunity in response to Flg22-induced ROS burst [68]. MKS1 is one of the target proteins of MPK4, which is similar to WRKY25 and WRKY33 TFs. In response to the pathogen infection process, plants produce phytoalexins, such as camalexin (3-thiazol-2-yl-indole), to enhance disease resistance. MAPKKKα/MEKK1–MKK4/MKK5–MPK3/MPK6 plays a key role in camalexin production [69]. AtMKK9 is also required for the activation of MPK3 and AtMPK6 to generate camalexin (Figure 2) [70]. A recent study also showed that the MAPKKK δ-1 (MKD1)–MKK1/MKK5–MPK3/MPK6-dependent signaling cascade is involved in the full immune responses against both *Pseudomonas syringae* pv. tomato DC3000 (*PstDC3000*) and *Fusarium sporotrichioides* infection [71]. In addition, OsMKK10-2-OsMPK6 responds to SA-mediated plant pathogen defense responses through the phosphorylation of WRKY45 [72], and OsRLCK185 (receptor-like cytoplasmic kinase) converts immune signals perceived by PAMP effector rice chitin elicitor receptor kinase 1 (OsCERK1) and activates the downstream OsMAPKKKε–OsMKK4–OsMPK3/6 pathway [73]. Studies have also shown that OsMKK3–OsMPK7–OsWRKY30 and OsMKKK43-OsMKK4–OsMPK6 can promote rice bacterial blight resistance [74,75,76], while OsMPK15 plays a negative role in *Magnaporthe oryza* and *Xoo* tolerance through the SA and JA signaling pathways [77]. The OsMKK2–OsMPK1 module positively regulates ROS-dependent blast disease (susceptibility)-related cell death in *M. oryzae* infection [78]. Moreover, the increased activity of the GhMKK6–GhMPK4 cascade can improve cotton’s (*Gossypium hirsutum*) resistance to *Fusarium oxysporum* [79]. In addition to the defense response to bacterial and fungal infection, MAPK cascades also participate in counteracting insect feeding. For example, the MKK3–MPK1/2/7 module participates in the inhibition of insect feeding (Figure 2) [51].

### 3.4. MAPK Cascades in Abiotic Stress

In *A. thaliana*, the best-studied MAPK cascades responding to abiotic stress are MEKK1–MKK2–MPK4/MPK6 (Figure 2) [80]. The target kinases MPK4 and MPK6 downstream of Arabidopsis MKK2 were isolated by functional complement of osmotic-sensitive yeast mutants, and MKK2 is also activated by MEKK1 induced by cold and salt stress in Arabidopsis protoplasts [80]. MEKK1 mRNA has massive accumulation in response to abiotic stress, such as low temperature, high salt, or mechanical damage. The overexpression of MKK2 resulted in sustained MPK4 and MPK6 activity and the up-regulated expression of stress-induced marker genes, thus enhancing plant tolerance to freezing damage and salt stress. By contrast, in *mkk2* plants, MPK4 and MPK6 activity was impaired and showed hypersensitivity to salt and cold stress. MPK6 plays a role in negatively regulating plasma membrane fluidity in the process of cold acclimation and controlling stomatal opening and closing under ozone exposure [81]. Further studies revealed that the MPK3/6–ICE1 (Inducer of CBF expression 1)–CBF (C-repeat-binding factor)–COR (cold-responsive) module plays a critical role in freezing acclimation. AtMPK3 and AtMPK6 directly phosphorylate AtICE1, affecting its transcriptional activity and thereby attenuating the binding ability of the AtCBF3 promoter [82,83]. In addition, MKK1 is activated by salt, drought, and mechanical damage stress and thus phosphorylated MPK4, suggesting that the MAPK cascade is involved in plant abiotic stress signals. Low temperature, low humidity, hyper-osmosis, salt stress, touch, and wounding can rapidly induce AtMPK4 and AtMPK6 activity, but their mRNA and protein levels are not changed, indicating that they regulate the signal mainly through post-transcriptional protein modification [84]. On the contrary, the mRNA of AtMPK1 increased under salt stress. The overexpression of rice DSM1 (drought-hypersensitive mutant 1; a class of Raf MAPKKK) can improve plants’ resistance to drought and hyper-oxygen stress [85]. Transgenic tobacco with exogenous overexpression of ZmMPK7 significantly increased resistance to osmotic damage by the ROS-mediated peroxidase (POX) defense system [86]. Moreover, wheat TaMAPK enzymes regulate the heat stress response by ROS-activated signaling sensors under heat stress [87]. Other studies have shown that AtMKKK18 responds to drought stress by activating downstream AtMKK3 [88], and overexpression of BnMPK1 can enhance the tolerance of *B. napus* to drought stress [89]. Similarly, maize ZmMKK1, rice OsMKKK63, and cotton GhRaf19 can respond to salt stress [90,91,92], while cotton GhMAP3K14–GhMKK11 –GhMPK31 and maize ZmMPK6–ZmWRKY104 are involved in drought stress [64,93]. More obviously, GhRaf4 and GhMEKK12 display a positive tolerance to drought stress [94]. In addition, other proteins may also participate in response to various environmental stresses with MAPK cascades. For example, it was reported that Raf could directly phosphorylate SnRK2s in response to drought/simulated drought stress [95,96]. OsPP2C27 directly dephosphorylates OsMPK3 and OsICE1 and thus plays a negative regulatory role under cold stress in rice [97]. However, banana (*Musa acuminata*) MusaMPK5 can respond to cold stress by phosphorylating the downstream NAC (NAM/ATAF1/2/CUC2) TFs MusaNAC042 and MusaSNAC67 [98]. 

### 3.5. MAPK Cascades in Cell Division and Differentiation

Scientists found that a class of MAPK cascade, nucleus- and phragmoplast-localized protein kinase 1 (NPK1)–NQK1/MEK1–NRK1/MPK1, is involved in the cell division process of tobacco [99,100]. During cell division, MAPKK kinase NPK1 specifically phosphorylates NtMEK1 by binding to two microtubule driving proteins (NPK1-activating kinase-like protein1, NACK1 (NPK1-activating kinesin-like protein 1), and NACK2, which belong to the kinesin-like protein (KLP) family, which control cells to form normal cell plates in late M (Figure 2) [101]. Similarly, Medicago MMK3 activity is enhanced by microtubules in membrane formation during mitosis anaphase. The three kinases of Arabidopsis, ANP1/ANP2/ANP3, orthologous genes of tobacco NPK1, are highly expressed in the active region of cell division and participate in the cell division process together with downstream MKK6/ANQ1 and MPK4 (Figure 2) [102]. They influence the structural organization of mitosis through reversible phosphorylation of MAP65 (Microtubule-binding protein 65) proteins [103]. In brief, the MAPK cascade controls microtubule continuous remodeling during cell plate formation and the phragmoplast period [104]. Moreover, YDA–MKK4/MKK5–MPK3/MPK6 regulates the inflorescence structure by promoting cell proliferation downstream of RLKs (receptor-like protein kinases) [105]. YDA and MPK6 can also affect the direction of cell division and cytoplasm movement in the main lateral roots [106]. Furthermore, Resveratrol (RSV)-mediated MPK-1 activation was found to prolong reproductive life and delay reproductive senescence by maintaining mitotic germ cells [107].

It was confirmed that YDA plays a critical role in cellular differentiation during early embryonic development and stomatal formation [37]. In *yda* mutants, the zygotes did not differentiate into suspensors, and the surviving mutant plants differentiated irregularly during epidermal development, resulting in the destruction of cell spacers in stomata and the formation of stomata clusters [37]. The transcript levels of CPFS (cell plate fusion site) markers [specifically microtubule binding protein *TANGLED1* (*TAN* ) and *phragmoplast orienting kinesin 1*(*POK1*)] were also deregulated [106]. The expression of *△N-YDA* leads to the excessive growth of stipe and inhibits embryonic development and stomatal formation. Reverse genetics showed that the downstream YDA genes MKK4/MKK5 and MPK3/MPK6 also have the function of regulating the stomatal phenotype. Similarly, root growth in the manner of the continuous division of cells in the apical meristem is also associated with the YDA–MKK4/MKK5–MPK3/MPK6 signaling cascade [108,109]. In addition, similar to the *yda* mutant, the zygotes of the *mpk3/mpk6* double mutant showed a similar phenotype of cell differentiation, suggesting that MPK3/MPK6 is downstream of YDA [110]. Another study also showed that only MKK4/5/7/9 of the ten Arabidopsis MKKs affect stomatal development [111]. Furthermore, another MAPK pathway may be involved in cell division; although details of microtubule-related substrates are somewhat elusive, these substrates may justify the role of MPK18 and the MAPK phosphatase PROPYZAMIDE HYPERSENSITIVE 1 (PHS1) in microtubule regulation [112]. 

## 4. Substrates Identification of MAPKs

### 4.1. Interaction Domains of MAPKs and Their Substrates

**Figure 3:**
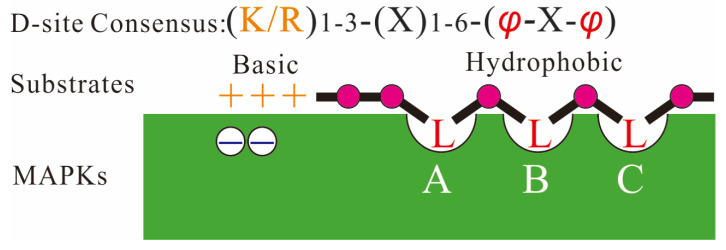
MAPKs interact with D-sites on substrates. The peptides display similar interactions with the two main regions of the MAPK: the hydrophobic groove, which has three side-chain docking pockets (**A**, **B** and **C**; **B** and **C** were earlier described as “-x-groove”), and the acidic region, known as the “common docking” (CD) site, which binds to the basic residues at the N-terminus of the docking motifs.

The specific docking interaction is the precondition of complex formation between MAPK and its connate activator, substrate, scaffold, or inactivator [113]. The docking interactions potentially contribute to the increased specificity of molecular recognition and enzymatic activity [114]. The CD domain of MAPKs is at the C-terminal region outside the catalytic domain, which is characterized by a cluster of negatively charged amino acids that can bind to the basic residues at the N-terminal D-site of the MAPK-interacting protein, such as substrates [115]. The D-site sequence is characterized by a cluster of basic residues and a hydrophobic motif, which usually harbors Leu (L), Ile (I), or Val (V), separated by one residue (R/K_1–3_-X1–6-φ-X-φ) (φ is any hydrophobic residue; Figure 3) [116]. The two hydrophobic residues at the distal end of the D-site in MAPKKs may determine the specificity of MAPK docking interactions [117]. All MAPKs have three (not two as originally thought) hydrophobic pockets on their surface, which together form a shallow “docking groove” that interacts with linear binding motifs LxLxL/I of their substrates [118,119] (Figure 3). Hydrophobic pockets exist in other variations, such as DEF (Asp-Glu-Phe), DFY (Asp-Phe-Tyr), and IYT (Ile-Tyr-Thr) motif, among others [17,120,121]. Therefore, proteins with similar D-site structures can be used as candidate MAPK substrates. 

### 4.2. General Strategy and Research Status of MAPK Substrate Identification

A large number of possible substrates for MAPK have been identified by Y2H, high-throughput protein array, and phosphoproteomic analysis [12,14,122]. In addition, the direct labeling of kinase substrates with ATP or kinase-sensitive analogs, such as ATP-γ-S, was reported [123]. Peptide library screening, similar to the protein microarray approach, is phosphorylated in vivo by the incubation of peptides rather than proteins. The phosphorylation sites were determined by proteomic analysis, and the possible substrates were inferred [124]. If a phosphorylation event is associated with MAPK activity, it is easier to determine the role of the kinases involved. In this case, the MAPK cascade-specific inhibitor U0126 is usually used to display the MAPK associated with phosphorylation events [125]. Both phosphoproteomic and affinity purification coupled with mass spectrometry (AP–MS) are screening methods based on MS. The former approach compares the overall levels of phosphorylation measured by quantitative phosphoproteomics in plants with and without MAPK activity. Proteins with significantly high phosphorylation can be considered as substrates for MAPK. For AP–MS, proteins that interact with labeled kinases and are extracted by affinity purification are identified by proteomics. Moreover, proximity labeling (PL) combined with MS can be used for more precise prediction in studying transient PPIs [126]. PL–MS has the potential to accurately examine hydrophobic interactions under native conditions in living cells and is based on the principle that proteins must be physically close [127]. MAPK–substrate interactions can also be detected by in silico prediction, immunoprecipitation, BN-PAGE (blue native polyacrylamide gel electrophoresis), co-localization of GFP (green fluorescent protein) or YFP (yellow fluorescent protein), SEC (size exclusion chromatography), BiFC (bimolecular fluorescence complementation), and FRET (fluorescence resonance energy transfer) [128,129].

**Figure 4:**
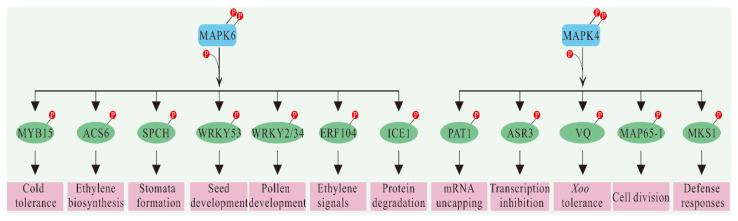
Schematic diagram for MAPKs and substrates in multiple plant signaling events. The examples of MPK4 and MPK6 are shown on the left and right panels, respectively. Moreover, SPCH, WRKY34, ICE1, and MYB15 were also phosphorylated by MPK3. The circled P indicates phosphorylation.

Although we identified a large number of possible substrates for MAPK through these methods mentioned above, only a few of them passed functional verification [130] (Figure 4). The first MAPK–substrate pair identified in plants was MPK6–ACS6. The phosphorylation of ACS6 by MPK6 enhances the stability and activity of ACS6, thus inducing ET biosynthesis [44]. SPCH (Speechless) is a class of basic helix-loop-helix (bHLH) TFs that initiate asymmetric cell differentiation to form stomata. Genetic analysis showed that *yda* mutants had a hyper-stomatal phenotype in the *spch* background, indicating that SPCH is downstream of the YDA–MKK4/MKK5–MPK3/MPK6 cascade [110]. In addition, YDA and HSP90.1 (heat shock protein 90.1) are epistatic and affect the phosphorylation of MPK6 and SPCH under acute heat stress [131]. MPK3/MPK6 phosphorylates several Ser/Thr residues of SPCH in vitro [132]. In vivo, mutations or deletions of SPCH phosphorylation sites can enhance stomatal formation activity. Therefore, MAPK phosphorylates SPCH, which makes the SPCH protein unstable and inhibits stomatal formation. MPK3 and MPK6 are functionally redundant in many physiological processes, but not all of them. For example, ET response factor 104 (ERF104) has been shown to be phosphorylated by MPK6 in vivo, while MPK3 has not [133], a process confirmed in FRET experiments. Flag22 stimulates MPK6 to phosphorylate ERF104, leading to its release, which subsequently regulates the expression of a target gene in the ET signaling pathway. Moreover, pollen-specific WRKY TF WRKY34 can be phosphorylated by MPK3/6 in the pollen double/triple cell stage [134]. Mutations of WRKY34 and WRKY2 lead to pollen defect phenotypes, including reduced pollen fertility, germination, and pollen tube development. Mutations at the MAPK3/6 phosphorylation site (Ser to alanine) at WRKY34 complement the *wrky2wrky34* double mutant phenotype, demonstrating the importance of the MPK3/6 phosphorylation site for preserving WRKY34 function. The ectopic dominant analysis confirmed that both MPK6 and WRKY34/WRKY2 belonged to the same signaling pathway. In addition, AtMPK3/6 also phosphorylates AtICE1 and AtMYB15, which induces rapid degradation of AtICE1 and inhibits the binding affinity of AtMYB15, which, in turn, weakens AtCBF3 transcription to enhance plant tolerance to freezing stress [83,135]. Furthermore, ZmWRKY104, a substrate of ZmMPK6, physically interacts with ZmMPK6 and is phosphorylated by it in maize [64]. OsWRKY53 is a direct substrate of the OsMKKK10–OsMKK4-OsMAPK6 cascade and regulates leaf angle and seed size control in rice [136].

The first substrate to be identified for MPK4 is MKS1. MKS1 may mediate MPK4 interaction with TFs to initiate plant defense responses [11]. Two subsequent studies proved that the phosphorylation of MKS1 by MPK4 is a prerequisite for the expression and activation of WRKY33 and PAD3 (PHYTOALEXIN DEFICIENT 3) [67]. In addition to MKS1, Y2H experiments also identified another MPK4 substrate, PAT1 (protein associated with Topoisomerase II 1), which is required for mRNA uncapping [10]. MAP65 is required for protein stability in the spindle central region during late cell division and is the target protein of the NPK1–NQK1–NRK1 pathway of the tobacco MAPK cascade and the homologous *A. thaliana* pathway. The phosphorylation of Thr-579 residues in the microtubule-binding domain of MAP65-1 by NRK1 can increase the affinity of MAP65-1 to microtubules. Conversely, it leads to overturning and instability of microtubules near the membrane formation body [137]. MAP65-1, MAP65-2, and MAP65-3 can all be phosphorylated by MAPK4 during cell division in Arabidopsis [138], while MAP65-1 is also phosphorylated by MPK6 in vivo [14,137]. Moreover, the newly identified MPK4 substrate is a transcription suppressor, ASR3 (ARABIDOPSIS SH4-RELATED3), which can enhance its DNA binding after phosphorylation by MPK4, thus inhibiting gene expression [125]. Some research has also indicated that OsVQ14 (valine-glutamine) and OsVQ32 act as substrates of the OsMPKK6–OsMPK4 cascade to improve rice tolerance to *Xoo* [76]. OsMPK4 phosphorylates OsVQ14 and OsVQ32 and can interact with them in vitro and in vivo.

## 5. Concluding Remarks and Future Perspectives

The unequal number of MAPK components and the bias to different stimuli make it difficult to find the interacting proteins of plant MAPKs. Gel kinase activity assay showed that not all MAPKs were active, especially TDY kinases, and inactive A- and B-class MAPKs are also usually ignored. A functional deletion mutant of the *MAPK* gene may be unable to accurately detect the gene function because of functional redundancy, and only some indirect evidence can be obtained. Similarly, MAPKs may have problems activating tags during post-translational modification. So far, MAPK substrates have mostly been reported only in Arabidopsis, and it is interesting to see how the knowledge we have gained from model plants can be better applied to crops. In addition, far fewer MAPKs substrates have been identified than predicted. As the number of plants with whole-genome sequencing increases, we can identify new substrates by comparative analysis of MAPK cascades between plant species. Quantitative phosphoproteomic also provides a very useful method for the identification of kinase substrates.

A major challenge for our next step is to confirm the phosphorylation function of MAPK–substrate interactions in biological processes. In fact, our functional characterization of substrates lags far behind large-scale MAPK characterization. Genetic, biochemical, and structural analysis methods have been applied. In addition, MAPK kinase N-terminal binding domain and scaffold proteins play an important role in controlling the specificity of cell signaling. Fortunately, MAPK cascades are very conserved in all eukaryotes, so techniques and achievements in animals and yeast can be applied to plant science. Another challenge is the complex cross-network between MAPK and other signaling pathways, in which different signals can be transmitted through the same pathway. There is also evidence that protein phosphorylation depends on post-translational modification to regulate substrate function, but the mode and network of action remain unclear. Therefore, the overall understanding of PPIs in MAPK signal transduction is still a direction and challenge for future research.

## Figures and Tables

**Table 1:** Composition and classification of MAPK cascades in Arabidopsis.

Family	Number	Group	Named Members	Active Sites *	References
MAPK	20	A	MPK3/6/10	TEY, TQY	[5,6]
		B	MPK4/5/11/12/13	TEY, MEY, TVY, TEC	
		C	MPK1/2/7/14	TEY	
		D	MPK8/9/15/16/17/18/19/20	TDY	
MAPKK	10	A	MKK1/2/6	S/T-X_5_-S/T	[5,20]
		B	MKK3	S/T-X_5_-S/T	
		C	MKK4/5	S/T-X_5_-S/T	
		D	MKK7/8/9	S/T-X_5_-S/T	
		E	MKK10	R-X_5_-S/T	
MAPKKK	80	MEKK	MEKK1, YDA, ANP1/2/3	G (T/S) Px (W/F) MAPEV	[21,22]
		Raf	MKD1, EDR1, CTR1	GTxx (W/Y) MAPE	
		ZIK	ZIK1	GTPEFMAPE (L/V/M)(Y/F)	

Note: * indicates all formation in plants, x represents any amino acid.

## Data Availability

Not applicable.
